# The Influence of Different Pretreatment Methods of Highland Barley by Solid-State Fermentation with *Agaricus sinodeliciosus* var. Chaidam ZJU-TP-08 on Its Nutrient Content, Functional Properties and Physicochemical Characteristics

**DOI:** 10.3390/jof8090940

**Published:** 2022-09-07

**Authors:** Biao Liu, Hongyun Lu, Qin Shu, Qihe Chen, Jinling Wang

**Affiliations:** 1 School of Forestry, Northeast Forestry University, Harbin 150040, China; 2 Department of Food Science and Nutrition, Zhejiang University, Hangzhou 310058, China

**Keywords:** highland barley, *Agaricus sinodeliciosus* var. Chaidam, solid-state fermentation, nutritional enrichment, biological activity

## Abstract

To enhance the nutritional value of highland barley (HB), this work investigated the effects of solid-state fermentation (SSF) by *Agaricus sinodeliciosus* var. Chaidam ZJU-TP-08 on nutrient content, phenolic components, antioxidant activities, and physicochemical characteristics of HB upon different pretreatments (germination, ultrasound and soaking). The results showed that germinated highland barley (GHB) exhibited higher levels of ergosterol (0.19 ± 0.01 mg/g) in all fermentation groups. The content of β-glucan was higher in the SSF-GHB, with an increase of 24.21% compared to the control. The content of total amino acids, dietary fiber, total phenols and flavonoids were higher in the fermentation HB pretreated by ultrasound, increasing respectively by 5.60%, 61.50%, 25.10% and 65.32% compared to the control group. In addition, the colonized HB exhibited excellent physicochemical characteristics, including increased water solubility index and decreased pasting characteristics. Herein, the nutritional value and the biological activities were enriched in the pretreated HB through SSF, indicating its potential application for nutrition-enriched functional foods.

## 1. Introduction

Known for high nutritional value, edible macrofungi are favored by consumers worldwide for their unique flavor and organoleptic properties [1]. Especially, macrofungi-derived secondary metabolites, including polysaccharides, phenolic compounds, and triterpenes, have a variety of health-promoting biological activities, contributing to a wide-accept research hotspot [2]. Ergosterol, as a triterpenoid compound found in the cell walls of fungi, has a large number of biological activities, including antibacterial, anti-inflammatory, anti-cancer, and cholesterol-lowering effects [3]. *Agaricus* *sinodeliciosus* var. Chaidam, a novel underground edible macrofungus from the Qinghai-Tibetan Plateau [4], was reported to have plenty of nutritional and bioactive compounds in the fruiting bodies and mycelia [5,6,7]. Due to the immature cultivation technology and remote habitat, the utilization and development of *A. sinodeliciosus* var. Chaidam are rarely reported. Therefore, it is necessary to explore an efficient method to obtain ergosterol from the cultured mycelia of *A. sinodeliciosus* var. Chaidam and expand its application in food processing.

Besides the fruiting body, mycelia and culture media in macrofungi are being explored as potential sources of bioactive compounds. Cultured mycelia was emerging as a promising alternative source of macrofungal bioactive compounds, mainly due to its shorter incubation time and easier culture conditions [8]. Converging lines of evidence suggested that edible mycelia colonized in grains by SSF have resulted in fermented nutrient-rich grain flours enriched with functional ingredients [9,10], which could be potential ingredients for new food formulations. Hence, the SSF of grains using macrofungal colonization is an effective method to improve the utilization of *A. sinodeliciosus* var. Chaidam and agricultural products.

Highland barley (HB), known as Qingke in China, is a special variety of barley (*Hordeum vulgare* subsp. *vulgare*) and a staple food for the people in Qinghai and Tibet provinces. Recently, HB has attracted increasing attention due to its unique nutrition values and bioactive compounds [11], especially β-glucan, which is a non-starch polysaccharide and has a great positive effect on human health, including cholesterol level reduction, CVD disease inhibition, tumors prevention, and immune enhancement [12]. However, it is difficult for most people to accept HB as a dietary food due to its rough taste, because approximate 98% of barley is devoted to the production of animal feed and wine processing, while only 2% is employed to the development of barley-based foods [13]. As previously reported, the increase in amino acids and active flavor compounds can enrich the taste in SSF. Meanwhile, various released enzymes could lead to hydrolysis of starch, cellulose and hemicellulose, thus improving the taste of grains [10,14].

To better enhance nutritional value and utilization of grains, the pre-treatments such as germination, ultrasound, and soaking are used frequently in the food processing industry [15]. Germination of grains is a non-chemical and simple method, and has received much attention for its physiological benefits, which increases the content of bioactive compounds including β-glucan and γ-aminobutyric acid [16]. The previous studies exhibited that consumption of germinated grains could decrease the risk of diabetes, obesity, cardiovascular disease and cancers [16,17]. Furthermore, an extensive review focused on the application of ultrasound in various food processing technologies, including bioactive compound extraction, food fermentation and food degradation, and it was confirmed that rice treated by ultrasound could enhance nutrition and improve texture [18]. Therefore, we made an interesting attempt to use the germinated, ultrasonic and soaked HB as the substrates for *A. sinodeliciosus* var. Chaidam mycelia fermentation to increase the availability of nutrients and biological activity.

Herein, different pretreated HBs by germination, ultrasound and soaking were colonized by *A. sinodeliciosus* var. Chaidam with the aim to obtain HB with enriched nutrient, enhanced bioactivities and physicochemical properties. In this study, we firstly provided HB colonized with *A. sinodeliciosus* var. chaidam ZJU-TP-08 to obtain bioactive cereal materials, and further detected the fermented nutrients including ergosterol, phenolic compounds and antioxidant activity, which contribute to potential health-promoting and future food application.

## 2. Materials and Methods

### 2.1. Microorganism, Culture Media and Cultivation Conditions

The edible macrofungus was isolated, purified and cultured from the wild *A. sinodeliciosus* var. Chaidam fruiting body. It was identified by morphological observation, physiological, and biochemical experiments and molecular biology, named as *A. sinodeliciosus* var. Chaidam ZJU-TP-08 and stored at China Center for Type Culture Collection (CCTCC M 2021511) [19]. Before the fermentation experiment, *A. sinodeliciosus* var. Chaidam ZJU-TP-08 was grown in potato dextrose agar (PDA) solid slant medium and incubated at 25 °C. The seed liquid cultivation was conducted in Erlenmeyer flasks (250 mL), containing 100 mL of the sterile cultivation medium and inoculated with 3 mycelial discs (1.5 cm in diameter). The flasks were covered with a layer of gauze and sterile cotton and then incubated in a dark incubator at constant temperature (150 rpm, 25 °C) for 4 days. The composition of the liquid fermentation medium (g/L) included 30 g of fructose, 8 g of yeast extract, 1.79 g of KH_2_PO_4_ and 1.18 g of MgSO_4_.

### 2.2. Pre-Treatment of HB

HB was purchased from a local farmers’ markets (Xining, China). Next, HB was washed 6–8 times with distilled water. Drained HB was then soaked in distilled water for 12 h at 25 °C, as the soaked group (SHB). Then, the soaked HB was used for sonication and germination respectively. Soaked HB was sonicated in distilled water for 2 h with an ultrasonic cleaner (40 KHz, Skymen Cleaning Equipment Shenzhen Co., Ltd., Shenzhen, China), as the ultrasonic group (UHB). Soaked HB was carried out at a constant temperature and humidity incubator (SaFe Experimental Instrument Technology Co., Ltd., Ningbo, China) at 25 °C and 85% relative humidity in the dark for 36 h, as germinated group (GHB). Then, GHB, UHB and SHB samples (each of 50 g) were distributed separately in glass flasks of 250 mL, then 1% CaCO_3_ and CaSO_4_ were added. The water content of solid fermentation substrate was around 35%. All the flasks were sterilized at 121 °C for 30 min. After cooling, the flasks with the sterile grains were inoculated in a vertical laminar flow cabinet.

### 2.3. Solid-State Fermentation Process

GHB, UHB and SHB were separately inoculated with 16 mL of liquid medium of *A. sinodeliciosus* var. Chaidam ZJU-TP-08 (SSF-GHB, SSF-UHB and SSF-SHB as the colonized groups). At the same time, GHB, UHB and SHB were separately inoculated with 16 mL of sterile water and were kept under the same cultivation conditions as control groups. The flasks were covered with a layer of gauze and sterile cotton and then incubated in a dark incubator at constant temperature (25 ± 1 °C) for 25 d. The media colonization was monitored daily by visual inspection. After fermentation, SSF-GHB, SSF-UHB and SSF-SHB were dried in an oven at 60 °C until to a constant mass. Then, they were ground in a Multi-functional crusher to obtain the colonized HB flours (standardized in 60 mesh). Non-inoculated GHB, UHB and SHB were submitted to the same procedures and referred to as control groups. The flour was stored in polyethylene packages at 4 °C until determination.

### 2.4. Mycelial Biomass Evaluation

Mycelial biomass of each type of fermented samples was estimated by extraction and quantification of ergosterol, as described previously with slight modifications [10]. Firstly, the biomass of *A. sinodeliciosus* var. Chaidam ZJU-TP-08 was obtained by submerged cultivation. The selected colonies were inoculated into ready-made basic liquid medium in 100 mL/250 mL conical flasks, incubated at 25 °C, and shaken at 150 r/min for 34 d. The content of the flasks were collected every 4 d. The mycelial biomass was separated by centrifugation (3220× *g*, 20 min, 4 °C) and lyophilized for subsequent extraction and quantification of ergosterol.

The extraction and determination of ergosterol were based on described literature previously, with slight modifications [10]. The samples (60 mesh) were extracted with methanol at a ratio of 1:10 (*m*/*v*) for 1 h at 25 °C, followed by 1 h of ultrasound treatment (40 KHz, Skymen Cleaning Equipment Shenzhen Co., Ltd., Shenzhen, China). The ergosterol extracts were collected after centrifugation at 3220× *g* for 15 min at 4 °C and filtered through 0.22 μm nylon filters. The ergosterol extracted from the mycelial biomasses and the flour samples (colonized and control groups) were determined in high-performance liquid chromatography (HPLC) system (Agilent Technologies Inc., Santa Clara, CA, USA) with VWD detector and reverse phase column Cosmosil 5 C18-MS-II (Nacalai tesque, Kyoto, Japan, 250 × 4.6 mm and particle size 5 µm), according to the method described previously with slight modifications [20]. Then, 20 μL of ergosterol extracts was injected into the system at 35 °C with mobile phase methanol: water (98:2, *v*/*v*), with a flow rate of 1 mL/min and detection wave at 282 nm. The identification of ergosterol was based on comparing the retention time with the standard solutions (0.001–1 mg/mL). The data obtained for the production of ergosterol in submerged medium were used to assay the mycelial biomass.

### 2.5. Nutritional Composition Evaluation

The analysis of the proximal composition of moisture, ash, crude protein, crude fat, crude fiber and dietary fiber of colonized and control groups was determined according to the method described previously [21]: Moisture (925.09), ash (incineration in a muffle furnace for 24 h at 550 °C) (no. 923.03), crude protein (total N × 6.25) (no. 992.23), crude fat (Soxhlet extraction method) (no. 920.39), crude fiber (sample digestion with diluted acid and alkali) (no. 962.09), and dietary fiber (no. 985.29). Total sugar and reducing sugar content were determined by the phenol-sulfuric acid colorimetric and the 3,5-dinitrosalicylic acid (DNS) method, as described previously [22]. The γ-amino butyric acid (GABA) content was determined according to the method described previously with slight modifications [23]. Samples of 0.5 g were weighed into a test tube, and then 5 mL of distilled water was added and oscillated for 2 h. The GABA contained in supernatant layer was isolated by centrifugation (3200× *g*, 15 min). Then, 0.6 mL of the boric acid buffer (0.5 M, pH 9.0), 2 mL of 5% re-steamed phenol solution and 0.2 mL of 10% sodium hypochlorite were added to 1 mL of the collected supernatant. The resultant mixture was boiled for 10 min in a water bath, then cooled in ice bath and followed by oscillation for 20 min until appearance of blue-green. Finally, 2 mL of 60% ethanol was added and the absorbance at 645 nm was recorded. The final quantification was based on a GABA calibration. The β-glucan content was quantified from flour samples (colonized and control groups) with a mixed-linkage β-glucan content assay kit (Suzhou Grace Biotechnology Co., Ltd., Suzhou, China) following the standard protocols mentioned in the kits.

### 2.6. Amino Acids Composition Detection

Prior to analysis, each sample (100 mg) of colonized and control groups was hydrolyzed with 4 mL (6 mol/L) hydrochloric acid for 24 h at 110 °C and filtered through 0.22 μm nylon filters. The amino acid composition was profiled using an automatic amino acid analyzer with a Na+ type cation exchange column (4.6 mm ID × 60 mm, 3 μm particles) and detection with a UV-V detector. Proline was detected at 440 nm and other amino acids at 570 nm. The color developer was ninhydrin/sodium acetate buffer, the buffer system was citrate buffer B1 (pH 3.2), B2 (pH 3.0), B3 (pH 4.0), and B4 (pH 4.9); the buffer flow rate was 0.4 mL/min; the column temperature was 55 °C, and the reaction temperature was 135 °C. Free amino acid content in sample solution was determined by external standard method. The results of the 17 amino acids investigated (lysine, valine, leucine, isoleucine, threonine, methionine, phenylalanine, histidine, arginine, tyrosine, serine, glutamic acid, proline, glycine, alanine, cystine, aspartic acid) were expressed as milligram of amino acids per gram of dry HB samples.

### 2.7. Total Phenolic and Flavonoid Content Detection

Free and bound phenolic compounds were extracted according to the method described previously [24]. Methanolic extracts were subsequently used for the total phenols, total flavonoids and antioxidant activity assays. Then, 1 g HB flour samples (colonized and control groups) was extracted three times with 20 mL of 80% methanol (*v*/*v*). For each extraction, the mixture was shaken for 30 min at room temperature in a shaker, then centrifuged for 15 min (3500× *g*), then the supernatant was vacuum evaporated to dryness at 45 °C, and finally the free phenolic content was reconstituted in 10 mL of methanol. After the extraction of free phenolic, the residue was extracted with 20 mL 2M NaOH solution and shaken at room temperature for 2 h; afterward, the mixture was acidified with HCl to pH 1.5–2.0, then centrifuged at 3500× *g* for 15 min. Hexane was used to extract lipids in the supernatant. The remaining mixture was extracted three times with ethyl acetate. The ethyl acetate fractions were pooled and evaporated to dryness. The bound phenolic content was reconstituted in 10 mL of methanol. All phenolic extracts were stored at −20 °C before analysis. Total phenolic content (TPC) and flavonoids content (TFC) were determined according to the method described previously [24]. TPC was calculated and expressed as milligram gallic acid equivalents (mg GAE/g). TFC was calculated and expressed as milligram rutin equivalents (mg RE/g).

### 2.8. Antioxidant Activity Assay

The total antioxidant capacity was conducted using the Total Antioxidant Capacity (T-AOC) assay kit (Solarbio Biochemical Assay Division, Beijing, China). The results were expressed as micromoles of Fe^2+^ equivalent antioxidant capacity per g of samples, using a Fe^2+^ calibration curve. The DPPH• and ABTS+• radical scavenging activities were determined based on the method described previously [25]. The DPPH ethanol solution (4.5 mL, 0.1 mmol/L) was mixed with 1.0 mL of samples and incubated in darkness (30 min). The absorbance of the solution was read at 517 nm. The DPPH scavenging activity was calculated as follows: scavenging effect (%) = (1 − A_1_/A_0_) × 100%, where A_1_ was the absorbance of the sample, and A_0_ was the absorbance of the blank control, the results were expressed as micromoles of Trolox equivalent antioxidant capacity (μmol TEAC/g). ABTS•+ working solution (4.0 mL) was mixed with 200 μL of samples and incubated in darkness (30 min). The absorbance of the solution was read at 734 nm. The ABTS•+ scavenging activity was calculated as follows: scavenging effect (%) = (1 − A_1_/A_0_) × 100%, where A_1_ was the absorbance of the sample, and A_0_ was the absorbance of the blank control. The results were expressed as micromoles of Trolox equivalent antioxidant capacity (μmol TEAC/g), the hydroxyl radical scavenging rate was determined according to the method described previously [26]. The reaction mixture contained FeSO_4_ (1 mL, 0.75 mM), 1,10-phenanthroline (1 mL, 0.75 mM), 1.5 mL of 0.15 M sodium phosphate buffer (pH 7.4), 1 mL of phenolic solution and 0.1 mL 0.3% H_2_O_2_ (1 mL, 0.01%, *v*/*v*) and 1 mL samples. After incubation at room temperature for 30 min, the absorbance of the mixture was measured at 536 nm. Hydroxyl radical scavenging (%) = (1 − A_1_/A_0_) × 100%, where A_1_ was the absorbance of the sample, and A_0_ was the absorbance of the blank control. The results were expressed as micromoles of Trolox equivalent antioxidant capacity (μmol TEAC/g).

### 2.9. Analysis of Physicochemical Characteristics

The bulk density (BD), water-holding capacity (WHC) and water solubility index (WSI) of the HB flour samples (colonized and control groups) were determined by a previously published method [27]. BD was calculated as the weight of sample per unit volume (g/mL). The pasting properties of the HB flour samples were analyzed according to previously reported method and used a Rapid Visco Analyzer (RVA Tecmaster, Perten, Hägersten, Sweden) [14]. Flour samples (3 g) in 25 mL of distilled water were added and measured with the RVA. The procedure was run as follows: 1 min at 50 °C, heated to 95 °C at a rate of 11.25 °C/min and held for 2 min, then cooled to 50 °C at 11.25 °C/min and maintained at 50 °C for 2 min. The peak viscosity (PV), tough viscosity (TV), breakdown viscosity (BV = PV−TV), final viscosity (FV), setback viscosity (SV = FV−TV) and peak temperature (PT) were recorded in this study.

### 2.10. Scanning Electron Microscope (SEM) Observation Morphological Features

The dehydrated samples were coated with gold–palladium in Hitachi Model E-1010 ion sputter for 4–5 min and observed in Hitachi Model SU-8010 SEM.

### 2.11. Statistical Analysis

All samples were conducted in triplicate. All experimental data were expressed in means ± SD and OriginPro software (2021 b, Southampton, MA, USA). One-way analysis of variance (ANOVA) followed by Duncan tests were conducted by SPSS statistics software (V.26.0, SPSS Inc., Chicago, IL, USA) to determine the significance level at *p* < 0.05. PCA analysis was implemented by using OriginPro software (2021 b, Southampton, MA, USA).

## 3. Results and Discussion

### 3.1. Effects of Different Pretreatments on Mycelial Biomass and Ergosterol Formation

**Figure 1:**
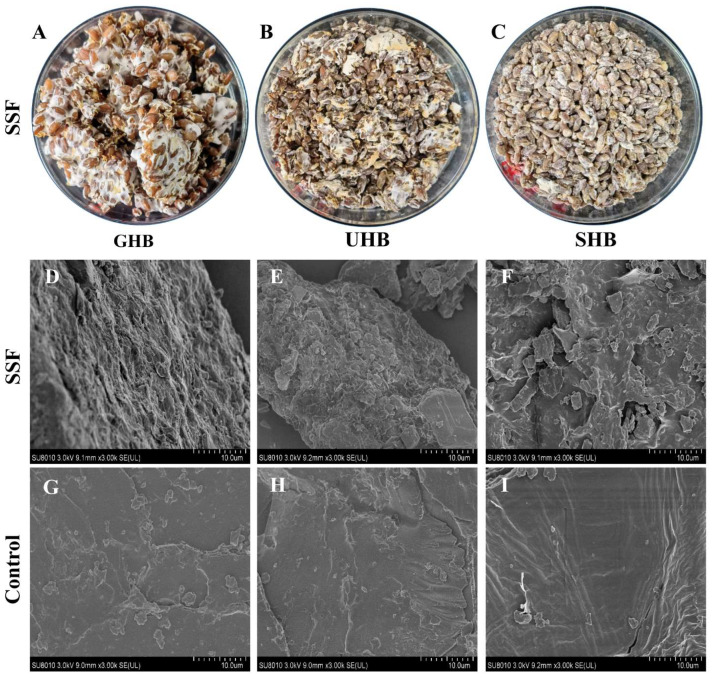
Appearance of solid-state fermentation highland barley. The letter (**A**–**C**) represents solid-state fermentation HB treated by germination (SSF-GHB), ultrasound (SSF-UHB), and soaking (SSF-SHB), respectively. Scanning electron microscopy of solid-state fermented and control highland barley. (**D**–**F**) indicate solid-state fermentation HB treated by germination (SSF-GHB), ultrasound (SSF-UHB), and soaking (SSF-SHB), respectively. (**G**–**I**) indicate germinated, sonicated, and soaked HB for control groups (GHB, UHB, SHB), respectively.

*A. sinodeliciosus* var. Chaidam ZJU-TP-08 was investigated in this study, which was able to colonize in HB pretreated by germination, ultrasound, and soaking process. Mycelia were observed on the surface and interior of the different cultivation media (Figure 1A–C). According to the results of SEM (Figure 1D–I), *A. sinodeliciosus* var. Chaidam ZJU-TP-08 mycelium colonized firmly onto GHB, UHB and SHB by SSF. However, the surface of unfermented HB was relatively smooth compared to SSF groups.

**Figure 2:**
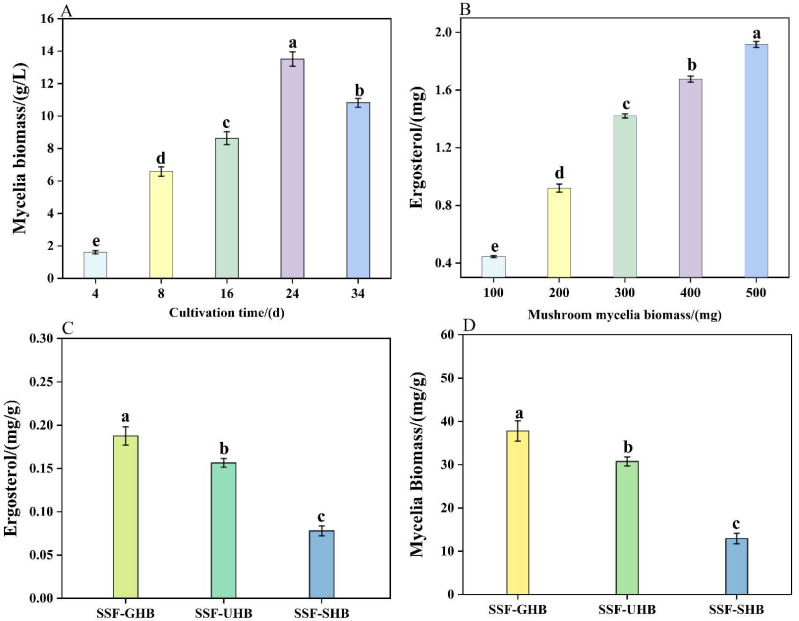
Ergosterol constituents of the highland barley colonized with *A. sinodeliciosus* var. Chaidam ZJU-TP-08 mycelia. (**A**): Mycelia biomass (g/L) produced by submerse cultivation during 34 d; (**B**): Ergosterol curve of mycelial at 24 d of cultivation; (**C**): Ergosterol determined (mg/g) of colonized Highland barley, the different colors of legends represent different group, (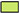) SSF-GHB, (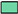) SSF-UHB, (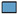) SSF-SHB; (**D**): Indirect determination of mycelial biomass (mg/g) of colonized Highland barley, the different colors of legends represent different group, (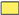) SSF-GHB, (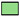) SSF-UHB, (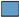) SSF-SHB. a–e: Values were determined in triplicate. Equal letters indicated that there were no significant difference at 5% (*p* > 0.05) in the parameter evaluated.

Due to the difficulty in separating the mycelial biomass from the grain, the indirect determination method was used with the ergosterol dosage to estimate the amount of mycelia present in the colonized HB. To quantify the changes in mycelial biomass during SSF, the correlation of ergosterol and mycelial biomass was performed. Clearly observed from Figure 2A,B, the quantitative correlation between mycelial biomass and ergosterol was performed (*y* = 0.0044*x* + 0.0214, *R*^2^ = 0.9913), indicating that the ergosterol can indirectly quantify the mycelial biomass in SSF. The ergosterol values was determined in different colonized HB and was shown in Figure 2C. The levels of ergosterol in the SSF-GHB, SSF-UHB and SSF-SHB were 0.19 ± 0.01, 0.16 ± 0.00 and 0.08 ± 0.01 mg/g, respectively. However, the level of ergosterol in unfermented HB was below the limit of detection. The mycelial biomass of colonized HB was presented in Figure 2D. Among all fermented samples, the highest amount of mycelial biomass was observed in the SSF-GHB (37.79 ± 2.35 mg/g).

### 3.2. Alterations in Nutritional Compositions

As shown in Table 1, the effect of *A. sinodeliciosus* var. Chaidam ZJU-TP-08 after colonization on GHB, UHB and SHB was further examined, including the chemical (the moisture, ash, crude fat, reduced sugar, total sugar) and nutritional compositions (crude fiber, dietary fiber, crude protein, β-glucan and GABA). The contents of crude protein in the SSF-GHB, SSF-UHB and SSF-SHB were 13.02 ± 0.06, 12.97 ± 0.22 and 12.95 ± 0.08 g/100 g. Compared to the crude protein in unfermented HB (the control groups), the protein content in SSF-GHB, SSF-UHB and SSF-SHB were increased by 5.20%, 5.40% and 6.00% (*p* < 0.05), respectively. As shown in Table 2, the contents of total amino acids in the SSF-GHB, SSF-UHB and SSF-SHB were 11.90 ± 0.10, 11.75 ± 0.04 and 11.74 ± 0.17 g/100 g. Compared to the total amino acid content in unfermented GHB, UHB and SHB, the contents of total amino acids in SSF-GHB, SSF-UHB and SSF-SHB were increased by 5.40%, 5.60% and 5.10% (*p* < 0.05), respectively. Particularly, the levels of valine, phenylalanine, glutamic acid, alanine and aspartic acid were increased among all fermentation groups (*p* < 0.05).

It has been reported that fermentation strategy was one of the best food processing techniques to increase the nutrition level of grains, such as protein [28]. Herein, the total protein content results demonstrated the potential of *A. sinodeliciosus* var. Chaidam ZJU-TP-08 for enriching proteins with HB. The most accepted explanation for the increase in protein content is that the microorganisms using substrate as carbon and energy sources to produce fungal protein, fungi can depolymerize the cell wall components and assimilate nitrogenous compounds from the substrates, altering the solubility of the proteins and improving the degradability of plant biomasses [10,14]. Fermentation by *Pleurotus ostreatus* (*Avena sativa*) for 336 h could increase oat protein levels [29], which was similar to our results. Similarly, the content of protein were increased 3–20% in fermented rice, due to the metabolic capacity of macrofungi during the fermentation process [30]. In the present study, the content of glutamic acid and aspartic acid were abundant in the SSF-GHB, SSF-UHB and SSF-SHB, and these amino acids are responsible for the production of fresh taste and are important for improving the taste of HB flour. A similar observation was noted in the brown rice, corn and wheat flour colonized by *A. blazei*, *P. albidus* and *A. fuscosuccinea* mycelia [10].

Dietary fiber plays an important role in promoting human health. In this work, the contents of dietary fiber in the SSF-GHB, SSF-UHB and SSF-SHB were 3.80 ± 0.12, 3.23 ± 0.06 and 3.21 ± 0.11 g/100 g. Compared to the dietary fiber in the non-fermented HB groups (GHB, UHB and SHB), the contents of dietary fiber in the SSF-GHB, SSF-UHB and SSF-SHB were increased by 53.80%, 61.50% and 51.20% (*p* < 0.05), respectively. According to a previous study [31], it was proven that the secretion and action of enzymes such as β-glucosidase, cellulase and xylanase rendered the decrease in dietary fiber during fermentation, which break down the polysaccharides of dietary fiber into smaller carbohydrates that serve as a source of energy and carbon for the metabolic processes. However, another research reported that fiber components were partially hydrolyzed and released by fungal enzymes (cellulase, xylanase, etc.) during fermentation process [32]. Therefore, the possible reasons for the increase in dietary fiber content were that the degradation of cellulose and hemicellulose and the formation of loose structures caused more soluble polysaccharides to be released during the SSF process. Meanwhile, it has been demonstrated that the total fiber content of fermented red quinoa seeds with *Neurospora intermedia* was increased by 73%, mainly attributing to the fungal biomass components [33]. Therefore, we speculated that the increase in dietary fiber content was attributed to the biomass component of the fungi in this study.

HB has been shown to be rich in β-glucan with many benefits [11,12]. Therefore, the changes of SSF on β-glucan content were also evaluated. The contents of β-glucan in the SSF-GHB, SSF-UHB and SSF-SHB were 4.72 ± 0.05, 4.29 ± 0.13 and 4.35 ± 0.21 mg/g, respectively. Compared to the β-glucan in the control groups (GHB, UHB and SHB), the contents of β-glucan in the SSF-GHB, SSF-UHB and SSF-SHB were increased by 24.21%, 20.51% and 18.53% (*p* < 0.05), respectively. The causes of elevated β-glucan can be attributed to two points of view. The increase in β-glucan content after fermentation was due to enhanced activity of enzymes secreted such as β-glucanases and carboxypeptidases, which caused degradation of total and insoluble β-glucan content into soluble β-glucan [34]. Furthermore, β-glucan was found in macrofungus mycelia [35]. We hypothesized that the increase in β-glucan after fermentation was possibly attributed to components of the fungal biomass.

GABA is a four-carbon non-protein free amino acid widely distributed in nature, which plays a role in regulating neuronal excitability throughout the nervous system [16]. The contents of GABA in the SSF-GHB, SSF-UHB and SSF-SHB were 0.88 ± 0.15, 0.78 ± 0.11 and 0.77 ± 0.12 mg/g (*p* > 0.05), respectively. The levels of GABA in the GHB, UHB and SHB (control groups) were 0.87 ± 0.14, 0.79 ± 0.08 and 0.83 ± 0.01 mg/g (*p* > 0.05), respectively. It was obvious that neither germination nor fermentation contributed significantly to the GABA content in this work. It has been reported that germination of grains could activate the corresponding enzymes to promote the biosynthesis of GABA, and brown rice germination promoted increased GABA content [16,17]. The exact cause for this change is still under examination in the future investigation.

### 3.3. Total Phenolic, Flavonoid Content and Antioxidant Activity

Phenolic compounds have been reported to help reduce the risk of various diseases and to promote the sensory characteristics of foods [17]. In this study, we evaluated the changes of SSF HB on phenolic compounds. Total phenols, total flavonoid and the total antioxidant activities upon different treatments of HB are presented in Table 3. For all fermentations, the levels of free, bound, and total phenolic compounds were superior to the unfermented substrate. Similarly, free, bound and total flavonoid exhibited the similar altered trends. The contents of total phenols in the SSF-GHB, SSF-UHB and SSF-SHB were 6.52 ± 0.22, 6.38 ± 0.47 and 5.58 ± 0.12 mg GAE/g, respectively. Compared to the control groups (GHB, UHB, and SHB), the contents of total phenols in the SSF-GHB, SSF-UHB and SSF-SHB were increased by 18.12%, 25.10% and 21.85%, respectively. The contents of total flavonoid in the SSF-GHB, SSF-UHB and SSF-SHB were 2.15 ± 0.07, 2.05 ± 0.04 and 1.87 ± 0.06 mg RE/g, respectively. Overall, the fermentation upon the three pretreated materials could improve the total phenolic and total flavonoid formation.

SSF increased the content of total phenolic was attributed to three aspects. Firstly, the phenolic compounds in grains mainly existed in the form of bound phenols, which could be released by alkali, acid or microbial enzymes [26]. For example, macrofungi had been reported to be capable for production of enzymes such as amylase, xylanase, cellulase and other enzymes that promoted the release of bound phenolic compounds [36]. The enzyme β-glucosidase had been described as being able to hydrolyze phenolic glycosides to release free phenolic acids [37]. It had been proven that α-amylase, xylanase and β-glucosidase produced during SSF of wheat by *Aspergillus oryzae* and *Aspergillus awamori* were highly correlated with the release of the phenolic compounds [38]. According to the previous investigation, total phenolic contents of brown rice, corn and wheat were enhanced after colonization by *A. blazei*, *A. fuscosuccinea* and *P. albidus* [10]. Secondly, microbial metabolism could modify the bioactive substances in grains, leading to the synthesis of new substances such as phenolic compounds. In addition, the increase in phenolic content may be due to the breakdown of grain cell walls by microbially produced enzymes and the subsequent release of phenolic compounds [39]. It has been shown that fungi possess extracellular enzyme-releasing systems for oxidizing lignin systems, which degrade lignin and open the benzene ring, ultimately increasing the free phenolic content [40]. Macrofungal degradation of the grain cell wall could be one of the reasons for enhanced levels of phenolic compounds, however, considering the quite limited proportion of the lignin in grain cell wall of highland barley. Thus, this pathway may not be the main origin of total phenolic levels in fermented highland barley. The release of phenolic compounds by enzymes alone during fermentation was limited. It was said the pathway of polyketide metabolism has been shown in *Monascus* strains, and the biosynthesis of polyketids was capable of generating polyphenols, and this might cause the increase in phenolic content [41].

SSF had been increasingly employed to increase the content of phenolic compounds in certain food products, thus giving rise to enhance antioxidant activity. A single antioxidant method could not fully reflect the antioxidant capacity of the sample. Thus, four antioxidant methods were used to investigate the antioxidant capacities of the free and bound phenolic extracts in the present study, including T-AOC, DPPH•, ABTS^+^• and hydroxyl radical scavenging activity. In this work, the levels of antioxidant in free and bound phenol extracted from SSF HB were significantly superior to those in the substrate without fermentation (*p* < 0.05), which was supported by previous findings [10,24,42,43]. Increased antioxidant properties of fermented grains may be inextricably linked to phenolic compounds. It has been demonstrated that soybean products fermented by SSF using *Trichoderma harzianum* showed intensive antioxidant activity than unfermented products, which was probably related to the markedly higher content of phenolic acids, flavonoids with more free hydroxyl groups achieved during SSF [44].

### 3.4. The Description and Characterization of Physicochemical Properties

The physicochemical properties are largely responsible for the final textural and sensory qualities of baked, steamed and boiled process of HB flours. Therefore, the effect of SSF on physicochemical properties of HB was evaluated in the present work. The bulk density (BD), water-holding capacity (WHC), water solubility index (WSI) and pasting property of HB are presented in Table 4. The capacities of WSI in the SSF-GHB, SSF-UHB and SSF-SHB were 26.13 ± 1.88%, 27.36 ± 0.26% and 22.10 ± 1.05%, respectively. The capacities of WSI of all fermented HB flour samples were increased, compared to control groups. However, the levels of BD and WHC were insignificant difference to those of the control groups (*p* > 0.05). Previous research studies had also pointed out that fermentation increased the capacity of WHC in the samples, but there was no effect on BD index [27]. RVA was frequently used to study the pasting, gelatinization and other physicochemical properties of flours [45]. In this study, all fermented samples were significantly reduced in the peak viscosity (PV), tough viscosity (TV), breakdown viscosity (BV), final viscosity (FV), setback viscosity (SV) and peak temperature (PT), compared to the control groups (*p* < 0.05). The reduction in the pasting properties of the fermented samples might be partly attributed to the degradation of starch by α-amylase, which was released in fungal metabolism. It was reported that the solid state fermentation reduced the pasting properties of brown finger millet [14].

### 3.5. Principal Component Analysis of Nutritional Constituents of Fermented and Unfermented HB

**Figure 3:**
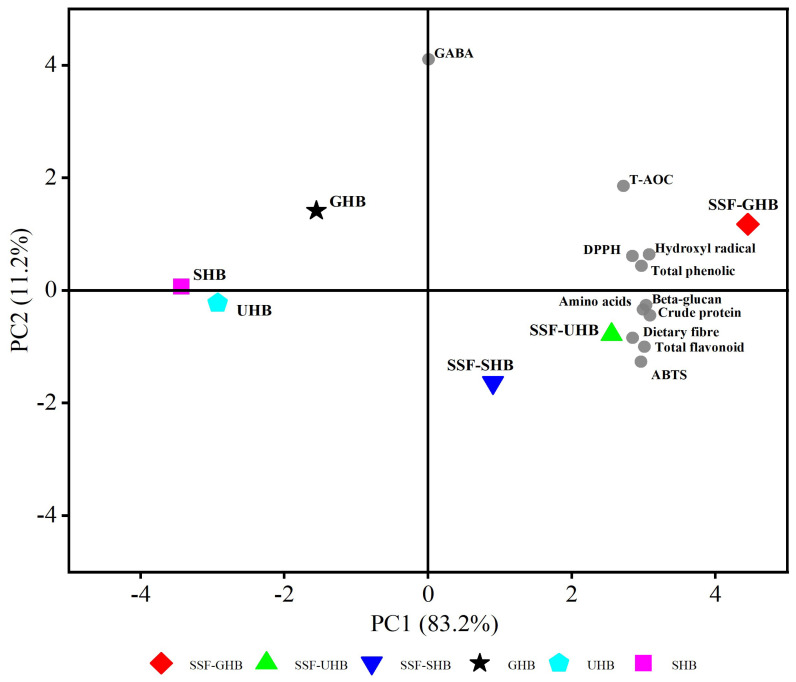
Nutritional constituents and biological activity of highland barley (the control groups and colonized groups with the mycelia of *A. sinodeliciosus* var. Chaidam ZJU-TP-08) were visualized by principal component analysis (PCA).

To highlight the key features of HB flour after SSF, principal component analysis (PCA) was performed, as the data show in Figure 3. Differences and similarities among the three SSF samples and their corresponding control groups in terms of eleven proximate compositions were visualized by PCA analysis, and the total variance (94.4%) was explained by the first two components, PC1 (83.2%) and PC2 (11.2%). SSF-GHB, SSF-UHB and SSF-SHB were located in the first and fourth quadrants, and all the proximate components (except for GABA) were distributed in the first and fourth quadrants, suggesting that SSF had a significant improvement in the nutrients, phenolic compounds and antioxidant properties of HB. However, GHB, UHB and SHB were located in the second and third quadrants, which showed insignificant correlation with the nutrients, phenolic components and antioxidant activities. In the previous study, principal component analysis (PCA) evaluated different solid-state fermentation media and the active components and antioxidant activities in mycelial fermentation products, indicating that solid-state fermentation could improve the active ingredients and antioxidant properties [46]. Similarly, our results of PCA analysis indicated that solid-state fermentation by *A. sinodeliciosus* var. Chaidam ZJU-TP-08 could enriched nutrients, phenolic components and antioxidant activities of HB.

## 4. Conclusions

To sum up, the solid-state fermentation with *A. sinodeliciosus* var. Chaidam ZJU-TP-08 was an effective method to enhance the nutrients, phenolic components and antioxidant activities of HB. In all of the fermentation groups, SSF-GHB exhibited higher levels of ergosterol and β-glucan with 0.19 ± 0.01 and 4.72 ± 0.05 mg/g, respectively. In addition, compared to UHB, SSF-UHB increased levels of amino acids by 5.60%, dietary fiber by 61.50%, total phenols by 25.10% and total flavonoids by 65.32%, which were the most significant increases among all fermentation groups. For all fermentations, the levels of antioxidant activities were superior to those in the substrate without fermentation. Meanwhile, the WSI of all fermentations was increased. However, the pasting characteristics were decreased after solid-stated fermentation, indicating their potential as functional ingredients in the development of instant products and other food applications. In view of the results obtained, GHB, UHB and SHB colonized by *A. sinodeliciosus* var. Chaidam ZJU-TP-08 are potential processing approaches in functional food development, and further studies are recommended to elucidate the mechanisms involved in the biotransformation process.

## Figures and Tables

**Table 1:** Nutritional constituents (dry matter) of the highland barley (the control groups and colonized groups with the mycelia of *A. sinodeliciosus* var. Chaidam ZJU-TP-08).

Parameters	SSF			Control		
	GHB	UHB	SHB	GHB	UHB	SHB
Moisture (g/100g)	12.38 ± 0.72 ^a^	12.63 ± 1.11 ^a^	11.57 ± 0.50 ^bc^	11.11 ± 1.08 ^bc^	10.16 ± 0.52 ^c^	10.06 ± 0.92 ^c^
Ash (g/100g)	4.36 ± 0.14 ^a^	4.03 ± 0.10 ^ab^	4.03 ± 0.06 ^ab^	4.01 ± 0.07 ^ab^	3.69 ± 0.19 ^b^	3.50 ± 0.15 ^b^
Fat (g/100g)	1.33 ± 0.05 ^d^	1.43 ± 0.06 ^cd^	1.49 ± 0.02 ^cd^	1.52 ± 0.04 ^bc^	1.68 ± 0.11 ^ab^	1.70 ± 0.08 ^a^
Reduce sugar (mg/g)	11.17 ± 0.68 ^a^	7.22 ± 0.49 ^b^	6.32 ± 0.26 ^b^	6.94 ± 0.43 ^b^	4.27 ± 0.29 ^c^	3.89 ± 0.25 ^c^
Total sugar (mg/g)	49.10 ± 1.50 ^a^	40.25 ± 0.31 ^b^	33.12 ± 0.35 ^c^	18.85 ± 0.57 ^e^	18.66 ± 0.29 ^e^	22.07 ± 0.78 ^d^
Crude fiber (g/100g)	2.68 ± 0.05 ^a^	2.51 ± 0.01 ^a^	2.68 ± 0.28 ^a^	1.78 ± 0.16 ^b^	1.57 ± 0.09 ^b^	1.94 ± 0.22 ^b^
Dietary fiber (g/100g)	3.80 ± 0.12 ^a^	3.23 ± 0.06 ^b^	3.21 ± 0.11 ^b^	2.47 ± 0.11 ^c^	2.00 ± 0.12 ^d^	2.13 ± 0.20 ^d^
Crude protein (g/100g)	13.02 ± 0.06 ^a^	12.97 ± 0.22 ^a^	12.95 ± 0.08 ^a^	12.38 ± 0.10 ^b^	12.30 ± 0.05 ^b^	12.23 ± 0.04 ^b^
Beta-glucan (mg/g)	4.72 ± 0.05 ^a^	4.29 ± 0.13 ^b^	4.35 ± 0.21 ^ab^	3.80 ± 0.15 ^c^	3.56 ± 0.24 ^c^	3.67 ± 0.15 ^c^
GABA (mg/g)	0.88 ± 0.15 ^a^	0.78 ± 0.11 ^a^	0.77 ± 0.12 ^a^	0.87 ± 0.14 ^a^	0.79 ± 0.08 ^a^	0.83 ± 0.01 ^a^

^a^, ^b^, ^c^, ^d^, ^e^: Values were determined in triplicate. Equal letters in the same row indicated that there were no significant difference at 5% (*p* > 0.05) in the parameter evaluated.

**Table 2:** Amino acids of the highland barley (the control groups and colonized groups with the mycelia of *A. sinodeliciosus* var. Chaidam ZJU-TP-08).

Amino Acids (g/100 g)	SSF			Control		
	GHB	UHB	SHB	GHB	UHB	SHB
Essential amino						
Lysine	0.43 ± 0.00 ^ab^	0.47 ± 0.04 ^a^	0.44 ± 0.00 ^ab^	0.45 ± 0.02 ^ab^	0.40 ± 0.00 ^b^	0.44 ± 0.02 ^ab^
Valine	0.64 ± 0.04 ^a^	0.71 ± 0.06 ^a^	0.68 ± 0.01 ^a^	0.57 ± 0.19 ^a^	0.63 ± 0.01 ^a^	0.61 ± 0.03 ^a^
Leucine	0.89 ± 0.05 ^a^	0.89 ± 0.03 ^a^	0.94 ± 0.14 ^a^	0.92 ± 0.03 ^a^	0.87 ± 0.06 ^a^	0.84 ± 0.09 ^a^
Isoleucine	0.53 ± 0.03 ^a^	0.54 ± 0.01 ^a^	0.56 ± 0.01 ^a^	0.52 ± 0.03 ^a^	0.55 ± 0.00 ^a^	0.52 ± 0.01 ^a^
Threonine	0.47 ± 0.04 ^a^	0.47 ± 0.04 ^a^	0.42 ± 0.03 ^a^	0.47 ± 0.06 ^a^	0.46 ± 0.01 ^a^	0.47 ± 0.01 ^a^
Methionine	0.18 ± 0.03 ^a^	0.15 ± 0.00 ^abc^	0.17 ± 0.01 ^ab^	0.15 ± 0.00 ^abc^	0.14 ± 0.00 ^c^	0.14 ± 0.00 ^bc^
Phenylalanine	0.74 ± 0.02 ^a^	0.69 ± 0.05 ^a^	0.71 ± 0.13 ^a^	0.70 ± 0.02 ^a^	0.67 ± 0.05 ^a^	0.68 ± 0.04 ^a^
Histidine	0.34 ± 0.00 ^a^	0.34 ± 0.01 ^a^	0.34 ± 0.01 ^a^	0.34 ± 0.01 ^a^	0.32 ± 0.01 ^a^	0.31 ± 0.00 ^a^
Non-essential						
Arginine	0.66 ± 0.01 ^a^	0.65 ± 0.01 ^a^	0.61 ± 0.04 ^a^	0.63 ± 0.02 ^a^	0.63 ± 0.02 ^a^	0.62 ± 0.02 ^a^
Tyrosine	0.61 ± 0.01 ^a^	0.61 ± 0.01 ^a^	0.56 ± 0.06 ^a^	0.63 ± 0.10 ^a^	0.60 ± 0.02 ^a^	0.62 ± 0.03 ^a^
Serine	0.30 ± 0.01 ^a^	0.28 ± 0.00 ^ab^	0.27 ± 0.01 ^bc^	0.26 ± 0.00 ^bcd^	0.25 ± 0.01 ^d^	0.26 ± 0.00 ^cd^
Glutamic acid	1.81 ± 0.16 ^a^	1.66 ± 0.07 ^a^	1.58 ± 0.19 ^a^	1.61 ± 0.05 ^a^	1.58 ± 0.07 ^a^	1.57 ± 0.13 ^a^
Proline	0.60 ± 0.01 ^a^	0.61 ± 0.02 ^a^	0.66 ± 0.06 ^a^	0.59 ± 0.02 ^a^	0.62 ± 0.08 ^a^	0.66 ± 0.10 ^a^
Glycine	0.70 ± 0.10 ^a^	0.67 ± 0.04 ^a^	0.69 ± 0.08 ^a^	0.64 ± 0.03 ^a^	0.63 ± 0.01 ^a^	0.63 ± 0.02 ^a^
Alanine	1.14 ± 0.11 ^a^	1.16 ± 0.08 ^a^	1.15 ± 0.20 ^a^	1.00 ± 0.11 ^a^	0.96 ± 0.05 ^a^	0.98 ± 0.06 ^a^
Cystine	0.33 ± 0.03 ^a^	0.33 ± 0.01 ^a^	0.35 ± 0.02 ^a^	0.32 ± 0.01 ^a^	0.32 ± 0.02 ^a^	0.31 ± 0.02 ^a^
Aspartic acid	1.64 ± 0.01 ^a^	1.52 ± 0.12 ^a^	1.62 ± 0.02 ^a^	1.50 ± 0.09 ^a^	1.51 ± 0.02 ^a^	1.51 ± 0.06 ^a^
Total	11.90 ± 0.10 ^a^	11.75 ± 0.04 ^a^	11.74 ± 0.17 ^a^	11.2 9± 0.19 ^b^	11.13 ± 0.02 ^b^	11.17 ± 0.09 ^b^

^a^, ^b^, ^c^, ^d^: Values were determined in triplicate. Equal letters in the same row indicated that there were no significant difference at 5% (*p* > 0.05) in the parameter evaluated.

**Table 3:** Total phenols, total flavonoids and antioxidant activity of the highland barley (the control groups and colonized groups with the mycelia of *A. sinodeliciosus* var. Chaidam ZJU-TP-08).

Biological Activity		SSF			Control		
		GHB	UHB	SHB	GHB	UHB	SHB
Total phenols (mg GAE/g)	Free	3.38 ± 0.06 ^a^	3.10 ± 0.26 ^ab^	2.60 ± 0.27 ^bc^	3.04 ± 0.12 ^ab^	2.46 ± 0.30 ^c^	2.21 ± 0.04 ^c^
	Bound	3.13 ± 0.08 ^a^	3.28 ± 0.21 ^a^	2.98 ± 0.15 ^a^	2.48 ± 0.10 ^b^	2.64 ± 0.04 ^b^	2.37 ± 0.03 ^b^
	Total	6.52 ± 0.22 ^a^	6.38 ± 0.47 ^a^	5.58 ± 0.12 ^b^	5.52 ± 0.16 ^b^	5.10 ± 0.35 ^bc^	4.58 ± 0.07 ^c^
Total flavonoids (mg RE/g)	Free	1.17 ± 0.04 ^a^	0.98 ± 0.07 ^a^	0.69 ± 0.09 ^b^	0.40 ± 0.03 ^c^	0.53 ± 0.21 ^bc^	0.48 ± 0.04 ^bc^
	Bound	0.94 ± 0.13 ^b^	0.86 ± 0.04 ^bc^	0.79 ± 0.03 ^cd^	1.09 ± 0.11 ^a^	0.71 ± 0.09 ^de^	0.67 ± 0.05 ^e^
	Total	2.15 ± 0.07 ^a^	2.05 ± 0.04 ^b^	1.87 ± 0.06 ^c^	1.49 ± 0.06 ^c^	1.24 ± 0.15 ^d^	1.15 ± 0.09 ^d^
Total antioxidant capacity (μmol Fe^2+^/g)	Free	2.71 ± 0.06 ^a^	2.19 ± 0.13 ^c^	2.17 ± 0.09 ^c^	2.34 ± 0.05 ^b^	2.09 ± 0.06 ^c^	2.02 ± 0.04 ^c^
	Bound	4.99 ± 0.31 ^a^	4.61 ± 0.15 ^b^	3.49 ± 0.11 ^d^	3.93 ± 0.12 ^c^	3.46 ± 0.10 ^d^	3.13 ± 0.08 ^d^
DPPH• radical scavenging (μmol TEAC/g)	Free	1.19 ± 0.05 ^a^	1.07 ± 0.06 ^b^	0.75 ± 0.03 ^d^	0.92 ± 0.03 ^c^	0.99 ± 0.06 ^bc^	0.57 ± 0.03 ^e^
	Bound	0.95 ± 0.07 ^a^	0.85 ± 0.03 ^b^	0.74 ± 0.04 ^c^	0.51 ± 0.04 ^d^	0.50 ± 0.03 ^d^	0.50 ± 0.05 ^d^
ABTS^+^• radical scavenging (μmol TEAC/g)	Free	2.63 ± 0.06 ^a^	2.40 ± 0.10 ^b^	2.19 ± 0.14 ^c^	1.99 ± 0.03 ^d^	2.19 ± 0.04 ^c^	2.47 ± 0.08 ^ab^
	Bound	2.24 ± 0.07 ^a^	2.22 ± 0.05 ^a^	2.13 ± 0.04 ^a^	1.43 ± 0.06 ^b^	1.25 ± 0.18 ^b^	1.35 ± 0.09 ^b^
Hydroxyl radical scavenging (μmol TEAC/g)	Free	1.22 ± 0.13 ^a^	1.31 ± 0.16 ^a^	0.99 ± 0.07 ^b^	0.92 ± 0.07 ^b^	0.74 ± 0.15 ^c^	0.68 ± 0.03 ^c^
	Bound	1.52 ± 0.09 ^a^	1.05 ± 0.06 ^b^	0.93 ± 0.05 ^c^	0.82 ± 0.05 ^d^	0.65 ± 0.06 ^d^	0.79 ± 0.04 ^e^

^a^, ^b^, ^c^, ^d^, ^e^: Values were determined in triplicate. Equal letters in the same row indicated that there were no significant difference at 5% (*p* > 0.05) in the parameter evaluated.

**Table 4:** Physical properties of the highland barley (the control groups and colonized groups with the mycelia of *A. sinodeliciosus* var. Chaidam ZJU-TP-08).

Parameters	SSF			Control		
	GHB	UHB	SHB	GHB	UHB	SHB
Functional properties						
BD (g/cm^3^)	0.56 ± 0.00 ^a^	0.55 ± 0.01 ^a^	0.55 ± 0.01 ^a^	0.54 ± 0.00 ^a^	0.54 ± 0.00 ^a^	0.54 ± 0.01 ^a^
WHC (g/g)	5.20 ± 0.18 ^a^	5.23 ± 0.00 ^a^	5.13 ± 0.35 ^a^	5.20 ± 0.53 ^a^	5.49 ± 0.10 ^a^	5.66 ± 0.09 ^a^
WSI (%)	26.13 ± 1.88 ^ab^	27.36 ± 0.26 ^a^	22.10 ± 1.05 ^abc^	21.41 ± 4.54 ^bc^	18.91 ± 1.01 ^c^	10.92 ± 1.38 ^d^
Pasting properties						
PV (cP)	293.00 ± 1.41 ^d^	140.50 ± 0.77 ^f^	209.50 ± 3.54 ^e^	652.00 ± 1.77 ^c^	829.00 ± 2.33 ^b^	1169.25 ± 1.06 ^a^
TV (cP)	285.00 ± 5.66 ^d^	140.00 ± 0.00 ^f^	205.00 ± 2.83 ^e^	608.00 ± 1.49 ^c^	775.00 ± 8.48 ^b^	1129.00 ± 5.66 ^a^
BV (cP)	5.50 ± 0.51 ^d^	0.50 ± 0.71 ^e^	6.50 ± 2.15 ^d^	44.00 ± 0.61 ^b^	54.00 ± 1.41 ^a^	41.00 ± 0.53 ^c^
FV (cP)	527.00 ± 0.71 ^d^	270.00 ± 2.02 ^f^	405.00 ± 20.51 ^e^	1003.00 ± 2.83 ^c^	1247.00 ± 17.68 ^b^	1698.00 ± 16.97 ^a^
SV (cP)	237.00 ± 2.73 ^d^	130.50 ± 2.09 ^f^	200.50 ± 17.68 ^e^	396.00 ± 4.24 ^c^	478.00 ± 26.16 ^b^	561.00 ± 11.31 ^a^
PT (°C)	n.d.	n.d.	n.d.	92.50 ± 2.12 ^a^	93.39 ± 1.51 ^a^	87.59 ± 1.90 ^b^
Peak time (min)	6.12 ± 0.11 ^b^	5.75 ± 0.09 ^c^	5.37 ± 0.04 ^d^	6.95 ± 0.07 ^a^	7.02 ± 0.02 ^a^	6.94 ± 0.01 ^a^

^a^, ^b^, ^c^, ^d^, ^e^, ^f^: Values were determined in triplicate. Equal letters in the same row indicate that there were no significant difference at 5% (*p* > 0.05) in the parameter evaluated. BD: bulk density, WHC: water-holding capacity, WSI: water solubility index, PV: peak viscosity, TV: trough viscosity, BV: breakdown viscosity, FV: final viscosity, SV: setback viscosity, PT: peak temperature.

## Data Availability

The study did not report any data.

## Sample Availability

Samples of solid state fermentation are available from the authors.

## Abbreviations

HB: Highland barley; GHB: Germinated Highland barley; UHB: Ultrasonic Highland Barley; SHB: Soaked Highland barley; SSF: solid-state fermentation; SSF-GHB: solid-state fermentation of GHB; SSF-UHB: solid-state fermentation of UHB; SSF-SHB: solid-state fermentation of SHB
